# Regulation of PD-L1 Expression by NF-κB in Cancer

**DOI:** 10.3389/fimmu.2020.584626

**Published:** 2020-11-25

**Authors:** Fabrizio Antonangeli, Ambra Natalini, Marina Chiara Garassino, Antonio Sica, Angela Santoni, Francesca Di Rosa

**Affiliations:** ^1^Institute of Molecular Biology and Pathology, National Research Council (CNR), Rome, Italy; ^2^Medical Oncology Department, Istituto Nazionale dei Tumori, Istituto di Ricovero e Cura a Carattere Scientifico, Milan, Italy; ^3^Department of Pharmaceutical Sciences, University of Eastern Piedmont, A. Avogadro, Novara, Italy; ^4^Humanitas Clinical and Research Center, Istituto di Ricovero e Cura a Carattere Scientifico, Milan, Italy; ^5^Department of Molecular Medicine, Laboratory Affiliated to Istituto Pasteur Italia, Sapienza University of Rome, Rome, Italy

**Keywords:** tumor associated macrophages, T cells, immune checkpoint inhibitors, tumor immunity, non-small-cell-lung cancer, tissue homeostasis, epithelial-mesenchymal transition, inflammation

## Abstract

Immune checkpoints are inhibitory receptor/ligand pairs regulating immunity that are exploited as key targets of anti-cancer therapy. Although the PD-1/PD-L1 pair is one of the most studied immune checkpoints, several aspects of its biology remain to be clarified. It has been established that PD-1 is an inhibitory receptor up-regulated by activated T, B, and NK lymphocytes and that its ligand PD-L1 mediates a negative feedback of lymphocyte activation, contributing to the restoration of the steady state condition after acute immune responses. This loop might become detrimental in the presence of either a chronic infection or a growing tumor. PD-L1 expression in tumors is currently used as a biomarker to orient therapeutic decisions; nevertheless, our knowledge about the regulation of PD-L1 expression is limited. The present review discusses how NF-κB, a master transcription factor of inflammation and immunity, is emerging as a key positive regulator of PD-L1 expression in cancer. NF-κB directly induces *PD-L1* gene transcription by binding to its promoter, and it can also regulate PD-L1 post-transcriptionally through indirect pathways. These processes, which under conditions of cellular stress and acute inflammation drive tissue homeostasis and promote tissue healing, are largely dysregulated in tumors. Up-regulation of PD-L1 in cancer cells is controlled via NF-κB downstream of several signals, including oncogene- and stress-induced pathways, inflammatory cytokines, and chemotherapeutic drugs. Notably, a shared signaling pathway in epithelial cancers induces both PD-L1 expression and epithelial–mesenchymal transition, suggesting that PD-L1 is part of the tissue remodeling program. Furthermore, PD-L1 expression by tumor infiltrating myeloid cells can contribute to the immune suppressive features of the tumor environment. A better understanding of the interplay between NF-κB signaling and PD-L1 expression is highly relevant to cancer biology and therapy.

## Introduction

The immune system relies on a complex balance between activating and inhibitory mechanisms to counteract infections and other threats while avoiding excessive tissue damage. Among the inhibitory molecules, a distinct set of inhibitory receptors and their ligands, collectively called “immune checkpoints,” has recently attracted a lot of attention for its relevance in cancer therapy, chronic infections, and autoimmune diseases (1). Programmed cell death protein-1 (PD-1) is a member of the CD28 family expressed by activated lymphocytes. PD-1 triggers immunosuppressive signals upon engagement by its ligands, i.e., PD-L1 (CD274 or B7-H1) and PD-L2 (CD273), which are members of the B7 family. While PD-L2 expression is largely restricted to antigen-presenting cells (APCs) and B1 lymphocytes, PD-L1 is expressed by APCs (mostly macrophages and dendritic cells), activated/exhausted T and B lymphocytes, and regulatory T cells (T_reg_), among others (2, 3). PD-L1 is also expressed by the cardiac endothelium, placenta, and pancreatic islets, with a possible role in maintaining immunological tolerance in these districts (4). Cancer cells can express PD-L1 and exploit the PD-L1-driven inhibitory pathway to their benefit as a key mechanism of immune evasion (5).

Return to the steady state at the end of immune response is tightly regulated (6, 7), and it is widely recognized that the PD-1/PD-L1 axis plays a central role in physiological immune homeostasis, contributing to the prevention of lymphocyte over-activation and immunopathology (1, 8). A variety of mechanisms have been involved in PD-1-mediated suppression of activated T lymphocytes, including exhaustion, inflammatory cytokine secretion inhibition, anergy, and apoptosis (8). PD-1 expression by antigen-responding T and B cells is tightly regulated, which allows for a stringent control of lymphocyte response (9, 10). Accordingly, PD-1 deficiency is associated with the development of autoimmune diseases (11, 12). PD-1 can be expressed also by γδ T cells, natural killer (NK) cells, and innate lymphoid cells (ILCs), which are circulating and tissue-resident lymphocytes involved in tissue repair and early responses against pathogens and cellular stress (13–16).

Regulation of PD-L1 expression and function takes place at different levels, as extensively reviewed by Sun and colleagues (17). Several mediators of inflammation are PD-L1 inducers, including TNFα, IFN-γ, IL-10, IL-17, and C5a (18–21). The JAK/STAT, RAS/MAPK, and PTEN-PI3K/AKT pathways are involved in the control of *PD-L1* gene expression *via* different downstream transcription factors, such as STAT1, STAT3, IRF1, IRF3, HIF-1α, MYC, JUN, BRD4, and NF-κB (22). The corresponding DNA-binding elements, except for IRF3, have been described on the PD-L1 promoter (23–32). Additional mechanisms of regulation include microRNA-mediated post-transcriptional inhibition (e.g., miR-513, miR-34a, miR-200, and miR-570) and the presence of a soluble form of PD-L1 (sPD-L1) in the blood, which possibly competes with the membrane-bound PD-L1 for binding to PD-1 (17, 33). Though only partially investigated, reverse signaling of PD-L1 has been reported in tumor cells and macrophages, resulting in pro-survival and inhibitory signals, respectively (34, 35). In addition to the PD-1/PD-L1 pair, a further interaction between PD-L1 and B7.1 (CD80) has been implicated in the inhibition of T-cell proliferation and cytokine production (36).

IFN-γ is one of the most studied PD-L1 inducers in tumors but PD-L1 expression does not necessarily mirror the IFN-γ signature (37, 38). NF-κB, a central player of inflammation and immunity, is emerging as a key positive regulator of PD-L1 expression. Notably, two recent studies, by using a CRISPR-Cas9-based wide screening approach, have identified NF-κB as a major determinant of cancer cell resistance against immune attack (39, 40). Considering the pivotal role played by PD-L1 for tumor cell immune evasion, the disclosure of the relationship between NF-κB signaling and PD-L1 expression is of great relevance (41). The present review gives an overview of the experimental works linking NF-κB to the regulation of PD-L1 expression in tumors. Furthermore, the implications of NF-κB-mediated control of PD-L1 expression for tissue homeostasis, cancer biology, and immune-therapy are discussed.

## NF-κB Among Inflammation, Immunity, and Cancer

NF-κB [nuclear factor kappa-light-chain-enhancer of activated B cells, discovered by Sen and Baltimore in 1986 (42)] is a transcription factor supporting host responses to cellular stress and immune responses to pathogens and other challenges. NF-κB can be composed of different dimers of the NF-κB family, activated downstream of multiple signaling pathways [for a comprehensive review on NF-κB see (43)]. Briefly, five proteins belong to the NF-κB family: p50, p52, p65 (RelA), RelB, and c-Rel; they are encoded by *NFKB1, NFKB2, RELA, RELB*, and *REL* genes, respectively. *NFKB1* and *NFKB2* codify for the p105 and p100 precursors, which are then processed to the active forms p50 and p52, respectively. The canonical (or classical) pathway leads to the activation of the p50/p65 (RelA) or p50/c-Rel heterodimers, while the non-canonical (or alternative) pathway leads to the activation of the p52/RelB heterodimer. The different heterodimers play distinct biological roles, controlling lymphoid organ development, immune activation, and cell survival (44–46). In healthy cells, NF-κB complexes are retained in the cytoplasm by inhibitory proteins belonging to the IκB family. Activating signals of the canonical pathway, which include TNFα, IL-1, and Lypopolysaccharide (LPS), cause IκB phosphorylation *via* IκB kinase (IKK). IKKα and IKKβ are the catalytic subunits of the multimeric IKK, which also includes the scaffold protein IKKγ (also named NEMO). Upon phosphorylation, IκB is ubiquitinated and targeted to degradation by the proteasome. This allows NF-κB's translocation to the nucleus where it regulates gene transcription by binding to the promoters of its target genes. Direct phosphorylation of p65 further enhances NF-κB nuclear translocation. In the non-canonical pathway, the inactive precursor of p52/RelB heterodimer is matured into its active form by IKKα phosphorylation and proteasomal processing upon NF-κB-induced kinase (NIK) activation by signals such as CD40L and lymphotoxin (47). A third atypical IKK-independent pathway is mainly triggered by hypoxia and UV radiation and leads to p50/p65 activation (48). NF-κB activation is regulated by several negative loops, including phosphorylation/de-phosphorylation and ubiquitination events. The NF-κB inhibitor IκBα is itself a transcriptional target of NF-κB. Different microRNAs (e.g., miR-146a, miR-302b) can contrast mRNA translation of proteins involved in the NF-κB cascade (49, 50).

Within the context of inflammation and immunity, NF-κB is activated downstream of the toll-like receptor (TLR)-MyD88 pathway that senses both pathogen-associated molecular patterns (PAMPs), such as LPS and other microbial products, and damage-associated molecular patterns (DAMPs), which are released by either stressed or dying host cells (51). NF-κB positively regulates the expression of inflammatory cytokines (TNFα, IL-1, IL-6), chemokines (CCL2, CCL5, CXCL8), adhesion molecules (VCAM1, ICAM1), angiogenic (VEGF), and anti-apoptotic factors (BCL-2, BCL-X_L_, FLIP), enzymes required for prostaglandin and NO synthesis (COX2, iNOS) (52). Furthermore, NF-κB is activated downstream of both the T-cell receptor (TCR) and B-cell receptor (BCR), sustaining the adaptive immune response (e.g., by controlling IL-2 expression) (53). NF-κB is also involved in NK-cell activation regulating IFN-γ production (54).

Within the context of cancer, NF-κB activation can support the neoplastic process (55, 56). One of the earliest pieces of evidence dates back to the discovery of the retroviral oncogene *v-Rel* (57). Furthermore, NF-κB can induce the transcription of the mitogenic factors MYC and Cyclin-D (58, 59). Finally, the AKT-mediated NF-κB activation, which frequently occurs in tumors, promotes cell survival and contributes to chemotherapy resistance of cancer cells (60). Remarkably, NF-κB activation in cells of the tumor immune infiltrate can have both anti-tumoral and pro-tumoral consequences depending on the immune cell type. On one hand, T cells, NK cells, and NKT cells require NF-κB for their anti-tumoral effector activity (61–63), but on the other hand, NF-κB sustains T_reg_ and myeloid-derived suppressor cell (MDSC) activity, resulting in pro-tumoral effects (64–66).

## NF-κB-Mediated Expression of PD-L1 by Cancer Cells

PD-L1 expression is often observed in tumors and correlates with aggressive behavior and poor prognosis. Whether PD-L1 is expressed by cancers cells, especially at the later stages of the disease, as negative feedback of a chronic inflammation process intertwined with cancer progression or as a consequence of cell selection by the immune system is still unclear. Certainly, PD-L1 expression confers a selective advantage to cancer cells, e.g., by enabling them to avoid host immune response by activated CD8 T lymphocytes and NK cells.

Considering the key role played by NF-κB in inflammation, immunity, and cancer, perhaps it is not surprising that NF-κB regulates PD-L1 expression in tumors, either directly at the transcriptional level or via indirect mechanisms. Different binding sequences for NF-κB have been described on the promoter of the *PD-L1* gene (Table 1). The canonical consensus for NF-κB DNA binding, named κB, consists of a nearly palindromic sequence, 5′-GGGRNWYYCC-3′ (where R: purine, Y: pyrimidine, W: adenine or thymine, and N: any base), which recently has been broadened to 5′-GGGNNNNNCC-3′ (32, 71, 72).

**Table 1:** Predicted binding sites for NF-κB on the *PD-L1* gene promoter.

**Consensus sequence**	**−5 −4 −3 −2 −1 0 +1 +2 +3 +4** **5^**′**^-G G G N N N N N C C-3^**′**^**	**N: any base**		
**Position**	**κB element**	**Validated by**	**Cell type**	**References**
From −387 to −378	GGGGG**A**CGCC	ChIP-PCR	TNBC	(67)
N/A	GGAAA**G**TCCA	Luciferase assay	Cervical cancer	(68)
N/A	GGAGC**G**TTCC	Luciferase assay		
From −1769 to −1760	GGCAA**A**TTCC		Macrophage	(69)
From −1293 to −1284	GGGAA**G**TCAC			
From −610 to −601	GGGAA**G**TTCT	ChIP-PCR		
From −75 to −66	GGAAA**G**TCCA			
From −606 to −597	N/A		Gastric cancer	(70)
From −238 to −229	N/A			
From −71 to −62	N/A	Luciferase assay		
From −49 to −40	N/A			

*Position refers to the transcription starting site. TNBC, triple-negative breast cancer; N/A, not available*.

**Figure 1:**
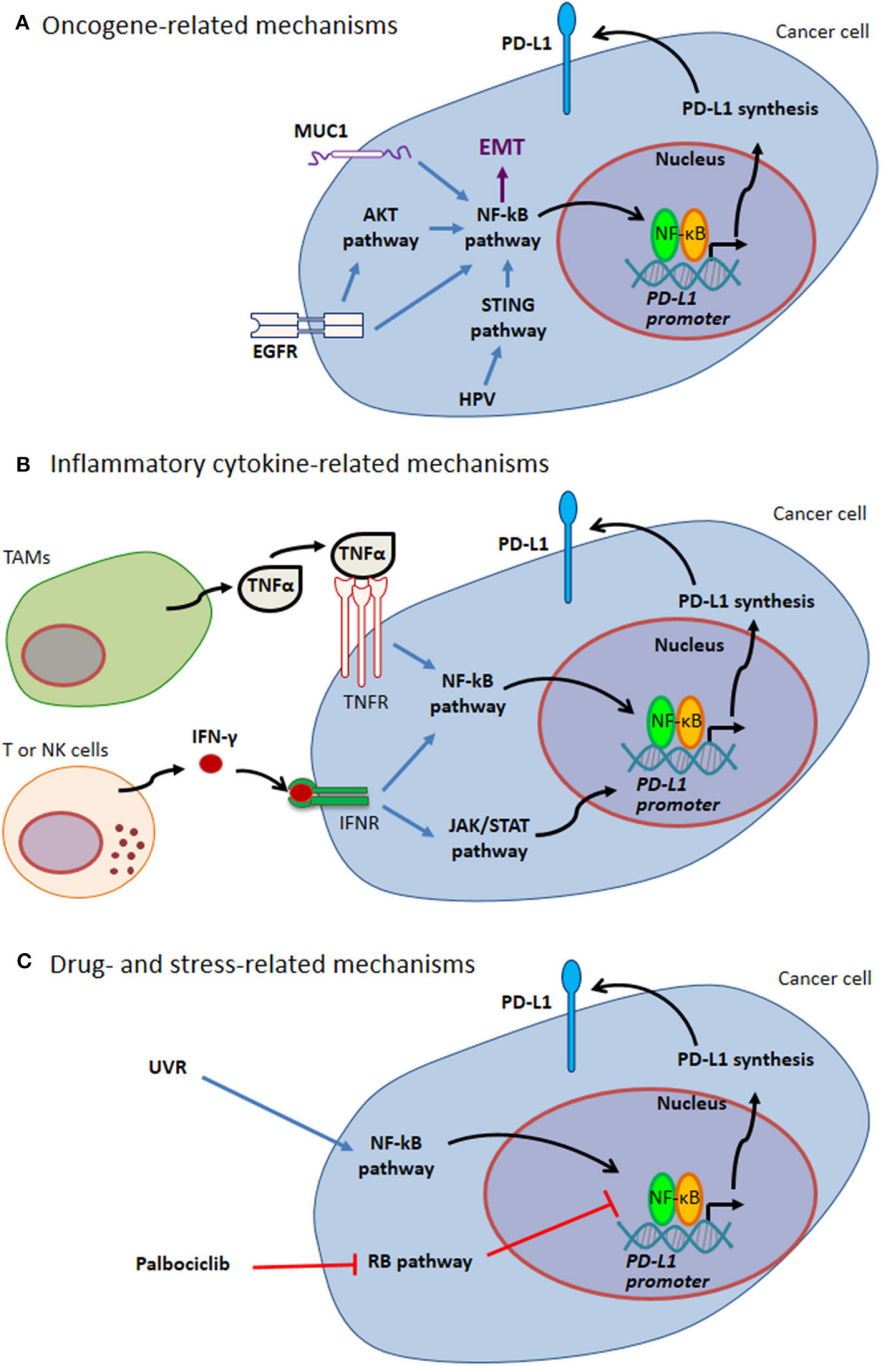
Mechanisms of PD-L1 expression through NF-κB. **(A)** Oncogene-related mechanisms. MUC1 and EGFR up-regulate PD-L1 expression by activating NF-κB pathway. These pathways are intertwined with EMT. HPV modulates PD-L1 expression triggering STING that in turn activates NF-κB. **(B)** Inflammatory cytokine-related mechanisms. Tumor-infiltrating immune cells can produce several cytokines regulating PD-L1 expression. Two well-known cytokines acting via NF-κB pathway are TNFα produced by TAMs and IFN-γ produced by tumor infiltrating T and NK cells. **(C)** Drug- and stress-related mechanisms. Different drugs act on NF-κB transcriptional activity (e.g., Palblociclib). Stress response to UVR activates NF-κB, thus mediating PD-L1 up-regulation. Blue arrows indicate activation of NF-kB pathway; black arrows indicate NF-κB-mediated PD-L1 up-regulation; red T-arrows indicate negative regulation. EGFR, Epidermal Growth Factor Receptor; EMT, Epithelial-Mesenchymal Transition; HPV, Human Papilloma Virus; IFN, Interferon; JAK, Janus Kinase; MUC1, Mucin 1; NK, Natural Killer; PD-L1, Programmed Cell Death Protein 1 Ligand; RB, Retinoblastoma; STAT, Signal Transducer and Activator of Transcription; STING, Stimulator of Interferon Genes; TAMs, Tumor Associated Macrophages; TNF, Tumor Necrosis Factor; UVR, ultraviolet radiation.

PD-L1 expression by cancer cells can be related to endogenous oncogenic pathways or oncogenic virus infection. In addition, PD-L1 expression by either cancer cells or tumor infiltrating cells can be driven by different kinds of exogenous stimuli, including cellular stress, e.g. stress induced by UV exposure or chemotherapy, as well as pro-inflammatory cytokines (such as TNFα and IFN-γ) in the tumor bed (Figure 1 and paragraphs below).

### Oncogene-Related Mechanisms

A direct link between the epidermal growth factor receptor (EGFR) and PD-L1 expression *via* NF-κB has been described in non-small-cell lung cancer (NSCLC), the most common lung cancer (73, 74). EGFR mutations associated with constitutive tyrosine kinase-mediated phosphorylation lead to NF-κB activation along with PD-L1 overexpression. Accordingly, the tyrosine kinase inhibitor gefitinib, approved for NSCLC treatment, reduces PD-L1 levels by inhibiting the NF-κB pathway. Notably, even wild-type EGFR induces PD-L1 up-regulation after stimulation with EGF (73). Mechanistically, EGFR triggers ERK, AKT, and IκBα phosphorylation, resulting in HIF-1α and PD-L1 accumulation (74). Activation of AKT is reported to increase HIF-1α protein translation, and it is known that the PD-L1 promoter contains an HIF-1α response element (27, 75, 76). As a consequence, EGFR activation in NSCLC promotes PD-L1 expression directly by phosphorylating IκBα and indirectly *via* HIF-1α. Furthermore, HIF-1α can support NF-κB pathway activation by driving *IKK*β gene transcription through a hypoxia response element present in the promoter and, at the same time, by directly inducing p65 (77, 78). Conversely, it has been reported that NF-κB can induce HIF-1α by binding directly to the HIF-1α promoter (79, 80). Thus, both HIF-1α and NF-κB pathways sustain PD-L1 expression and reinforce each other.

Overexpression of the oncoprotein Mucin 1 (MUC1) is correlated to NF-κB-mediated PD-L1 expression in both NSCLC and triple-negative breast cancer (TNBC) (81, 82). MUC1, through its MUC1-C subunit, activates the PI3K/AKT, MAPK, β-catenin/MYC, and NF-κB pathways (83). NF-κB activation by MUC1 occurs, at least, through three different mechanisms: (i) binding to p65, thus forming a p65/MUC1 transcriptionally active complex (84); (ii) binding to IKKβ-IKKγ complexes, which triggers IκBα phosphorylation (85); and (iii) associating with TGF-β-activated kinase-1 (TAK1), which phosphorylates IKKβ on Ser181 (86). Once activated, NF-κB enhances the expression of MUC1, thus creating a self-sustaining loop. At the same time, MUC1, *via* the YAP/β-catenin pathway, mediates the induction of the WNT target gene *MYC* (87). PD-L1 promoter contains both an E-box sequence (CAGCTT) for MYC binding at positions from −164 to −159 nt and a p65-binding site (GGGGGACGCC) at positions from −387 to −378 nt upstream of the transcription-starting site (67) (Table 1). Hence, MUC1 can induce the expression of PD-L1 triggering the occupancy of its promoter by both p65 and MYC.

It is remarkable that the signaling of PD-L1 induction shares important elements with that occurring during epithelial-mesenchymal transition (EMT), a tissue remodeling process typical of advanced epithelial cancers (88). PD-L1 expression is observed during EMT in NSCLC (89). Hypoxia and chronic inflammation are the main drivers of EMT, which is largely mediated by TGF-β1, TNFα, HGF, EGF, and PDGF (90, 91). Interestingly, PD-L1 expression is not regulated by the EMT-specific transcription factor SNAIL, but it involves a non-canonical NF-κB signaling. In particular, TNFα activates IKKε, which leads to p65 recruitment on the PD-L1 promoter. Concomitantly, TGF-β1 reduces DNA methyl transferase-1 (DNMT1) recruitment on the PD-L1 promoter, causing its de-methylation and thus enhancing its transcription. Notably, TNFα and TGF-β1 withdrawal reverts the EMT phenotype and, at the same time, abolishes PD-L1 expression (89). The interconnection between EMT and PD-L1 is evident in the MUC1 signaling with NF-κB as the matchmaker. NF-κB not only drives PD-L1 expression, but also the expression of ZEB1, a transcription factor that is able to mediate EMT (92). ZEB1, in turn, suppresses the transcription of miR-200, an inducer of epithelial differentiation, thus enhancing EMT and PD-L1 expression (93).

Taken together, these findings indicate that PD-L1 expression is a feature of EMT in cancer. Considering that (i) EMT occurs physiologically during embryonic development and (ii) PD-L1 expression can be enhanced by OCT4 and SOX2, two master “stemness” transcription factors driving the expression of genes necessary for the stem cell phenotype (94), it is tempting to speculate that NF-κB drives PD-L1 expression in the context of a general mechanism that has evolved to protect mesenchymal cells and stem cells from immune attack during physiological development.

It is now emerging that PD-L1 not only mediates negative feedback in the context of immune response, but it is also a component of a homeostatic response of epithelial cells to stress (95). In this case, PD-L1 expression can be linked to cellular conditions such as proliferation, adhesion, and migration (22). Supporting this consideration, MUC1, whose role in mammalians is to protect epithelial layers by forming a mucous barrier, senses cell stress and transduces signals that are able to activate the NF-κB pathway, which leads to PD-L1 expression (96). It is therefore convincing that NF-κB-mediated expression of PD-L1 takes part in cancer biology by echoing a process evolved to restore tissue integrity during the epithelial stress response.

Oncogenic viruses are also PD-L1 inducers. For example, infection by papilloma virus promotes PD-L1 expression in cervical cancer cells. Interferon-inducible 16 (IFI16) acts as viral DNA sensor and activates stimulator of IFN genes (STING), leading to TANK-binding kinase-1 (TBK1) activation that, in turn, initiates a cascade signaling that is able to recruit p-p65 on the PD-L1 promoter. The STING/TBK1 pathway activates both IRF3 and NF-κB, with NF-κB mediating a major contribution to *PD-L1* gene transcription (68). PD-L1 expression is also observed in NK/T cell lymphoma infected by Epstein-Barr virus. Both MAPK and NF-κB pathways are involved in PD-L1 induction in these cells (97).

### Inflammatory Cytokine-Related Mechanisms

NF-κB regulates PD-L1 expression downstream of inflammatory cytokine-induced pathways in the tumor microenvironment, contributing to linking together two immune-related hallmarks of cancer, i.e., tumor-promoting chronic inflammation and immune escape (98). IFN-γ and TNFα will be considered here in more detail.

IFN-γ is one of the most studied PD-L1-inducer inflammatory cytokines, which is produced by highly activated T and NK cells infiltrating the tumor (38, 99). Although it is well-established that IFN-γ signals *via* JAK/STAT (100), there is evidence that IFN-γ can also activate the NF-κB pathway, which in turn mediates PD-L1 up-regulation. For example, in melanoma cells, IFN-γ inducible expression of PD-L1 is linked to the activity of p50 and p65, and not to the activation of the interferon-related STAT factors (101). Moreover, inhibitors of the bromodomain and extra terminal (BET) proteins, a class of epigenetic regulators with immunomodulatory activity (102), reduce PD-L1 expression by inhibiting p50 transcription (103). Transcriptional up-regulation of *PD-L1* gene by NF-κB occurs also in clonal blasts from myelodysplastic diseases treated with IFN-γ and TNFα (104). Notably, also type I IFN, which can be produced by many types of cell stimulated by either PAMPs or DAMPs, is a PD-L1 inducer (105).

TNFα, which can be released by activated tumor associated macrophages (TAMs), is a major driver of inflammation and one of the main inducers of NF-κB in tumor microenvironment. It has been mentioned above that TNFα drives EMT and regulates PD-L1 expression (see previous paragraph). Apart from EMT, TNFα, IL-17, or a combination of both, can induce PD-L1 up-regulation *via* NF-κB (20). Moreover, Lim and colleagues have shown that TNFα-activated NF-κB can regulate PD-L1 post-transcriptionally through an indirect way (106). TNFα binds TNFR on cancer cells and induces, among other pathways, a signaling cascade that promotes p65 nuclear translocation *via* IKKβ. In the nucleus, p65 trans-activates the *COPS5* gene by binding to its promoter. *COPS5* codifies for COP9 signalosome 5 (CSN5), which is the catalytic subunit of a large multiprotein complex with deubiquitination activity (107). CSN5 is able to interact and deubiquitinate PD-L1 protein, increasing its stability and consequently its surface expression. The biological relevance of this regulatory mechanism is confirmed by the positive correlation observed in breast cancer specimens between p-p65, CSN5, and PD-L1 expression as well as the inverse correlation with granzyme B, a cytotoxic lymphocyte effector molecule (106). Notably, TNFα, through the activation of the NF-κB pathway, also up-regulates CSN2, which, by blocking the ubiquitination of the transcription factor SNAIL, promotes tumor invasiveness (108). Therefore, the inflammatory cytokine TNFα coordinates both EMT and tumor immune evasion by using NF-κB signaling (see also the previous paragraph). This pathway is negatively regulated by curcumin, a natural anti-inflammatory compound that is known to inhibit NF-κB signaling as well as CSN5 activity (109, 110). Accordingly, it has been shown that curcumin reduces TNFα-mediated PD-L1 stabilization (106).

### Drug- and Stress-Related Mechanisms

Drugs currently used in the clinic or in pre-clinical studies can influence PD-L1 expression *via* NF-κB, also influencing epigenetic regulation. Epigenetic events, changing the chromatin structure *via* methylation or acetylation/deacetylation, modulate gene expression. For example, taxolo (named also paclitaxel) and gemcitabine induce transient expression of PD-L1 mRNA in ovarian cancer by up-regulating p65, even in the absence of IFN-γ signaling (111). NF-κB nuclear activity is regulated by reversible acetylation/deacetylation of p65 operated by HDAC3 (112). As a result, two histone deacetylase (HDAC) inhibitors, namely resminostat and entinostat, affect NF-κB mediated PD-L1 expression (113).

Palbociclib, a recently developed CDK4/6 inhibitor, promotes PD-L1 protein stabilization and increases *PD-L1* gene transcription (114, 115). The latest effect is due to an indirect mechanism involving the retinoblastoma protein (RB), which acts as a negative regulator of NF-κB functions, as follows. Hyper-phosphorylated RB (phosphorylation at S249 and T252) specifically interacts *via* its N-terminal portion with p65 in the nucleus and, in this manner, blocks NF-κB transcriptional activity, including the NF-κB-dependent transcription of PD-L1. Inhibition of the RB pathway by palbociclib induces the hypo-phosphorylated status of RB, thus enhancing NF-κB-mediated PD-L1 transcription (115).

Solar ultraviolet radiation (UVR) is a common environmental stress for the skin and is largely involved in the carcinogenesis of skin cancers. Besides the well-known mutagenic effects, UVR can establish an immunosuppressive environment by different mechanisms, including CTLA-4 and PD-L1 up-regulation (116). PD-L1 transcriptional up-regulation in melanocytes and melanoma cells has been linked to the transcriptional activity of either NRF2, a regulator of antioxidant proteins, or NF-κB (117, 118). Regarding NF-κB, UVR B exposure causes in keratinocytes and melanocytes the subcellular translocation from the nucleus and release outside the cell of the high mobility group box 1 protein (HMGB1), an early stress response DAMP. HMGB1 acts in an autocrine and/or paracrine fashion and binds the receptor for advanced glycation endproducts (RAGE) leading to downstream kinase TBK1 activation. TBK1, and not TAK1, is involved in starting the NF-κB cascade after UVR B exposure even though TAK1 has been shown to play a role in the DNA-damage induced NF-κB signaling (119). TBK1 phosphorylates its canonical target IRF3 and IKKβ, which, by phosphorylating IκBα, removes its inhibition on p65. PD-L1 promoter contains two putative IFN-stimulated response elements (ISRE) along with NF-κB binding sites. Once activated, p65 forms the canonical p50/p65 heterodimer but also interacts with IRF3 itself forming a complex that is recruited to PD-L1 promoter at the NF-κB binding sites, thus starting *PD-L1* gene transcription. In agreement with PD-L1 up-regulation, and possibly mediated also by additional mechanisms, melanoma cells show a reduced susceptibility to CTL-dependent cytotoxicity after UVR B treatment (118).

## NF-κB-Mediated Expression of PD-L1 by Tumor Infiltrating Macrophages

Chronic inflammation can pave the way to tumor onset and cancers are often embedded in an inflammatory microenvironment that enhance tumor progression (120). In this context, investigating the regulation of PD-L1 expression by tumor infiltrating cells can shed light on the link between chronic inflammation, tumor progression, and immune escape. TAMs are key cellular players of the tumor infiltrate that can regulate the inflammatory process and, at the same time, act as antigen-presenting cells for CD4 T lymphocytes. Macrophages have heterogeneous phenotypes, ranging from classical M1 to alternative M2 cells, which represent extremes in a continuous spectrum of activation states, with M1 cells having tumoricidal activity and M2 cells favoring tumor progression (121, 122). In addition to the identification of PD-L1^+^ TAMs (123), PD-L1 has been found highly expressed in MDSCs, a population of tumor-infiltrating myeloid cells implicated in inhibition of other cells of the immune system (124, 125), and a MDSC molecular program linked to NF-κB activation has been related to PD-L1 expression (126).

NF-κB can directly regulates PD-L1 expression in macrophages and other myeloid cells stimulated by inflammatory cytokines (IL-12, IFN-γ), PAMPs, and DAMPs (69, 107, 127, 128). LPS, a prototypical PAMP, can stimulate PD-L1 expression by macrophages via TLR signaling. Indeed, within 1 h after LPS sensing, p65 translocates to the nucleus where it binds to the PD-L1 promoter, inducing *PD-L1* gene transcription independently of AP-1 and IRF3, two canonical transcription factors activated by LPS-TLR4 signaling (69). The mechanism whereby LPS promotes *PD-L1* gene expression through the transcriptional activity of NF-κB links inflammation to its control, and it is not restricted to macrophages but also occurs in tumors, as demonstrated in gastric cancer cells (70). Furthermore, melanoma-derived extracellular vesicles carrying Heat Shock Protein (HSP)-86, a typical DAMP, can stimulate PD-L1 expression by myeloid cells *via* TLR4 signaling. Interestingly, a strong NF-κB activation is observed in the immortalized myeloid suppressor cell line MSC-2 stimulated with the extracellular vesicles, and PD-L1 up-regulation is reduced in a dose-dependent manner by the NF-κB inhibitor Bay11-8082 (128).

Despite a direct role for NF-κB in the expression of PD-L1 by macrophages has been demonstrated in response to inflammatory cytokines, NF-κB activation in TAMs is the result of the combined action of both microenvironmental signals and microphysiological conditions (i.e., hypoxia, glucose levels, and pH), and accumulating evidence indicates that different activation states of NF-κB regulate functions and phenotypic heterogeneity of TAMs (121). Moreover, along with tumor-promoting functions, TAMs and monocytic MDSCs share similar molecular traits, such as nuclear accumulation of p50 NF-κB inhibitory homodimer, which drives M2 macrophage polarization and suppressive activity (66, 129, 130). The nuclear accumulation of p50 homodimer hinders the expression of inflammatory cytokines (TNFα, IL-1, IL-6) while increasing anti-inflammatory cytokines (IL-10, TGFβ) and chemokines (CCL17, CCL2), being therefore essential for the resolution of the inflammatory response (131). It thus appears that tumors co-opt transcriptional mechanisms guiding the resolution of inflammation, to promote cancer development. Inhibition of classical NF-κB activation in TAMs has been also observed in response to the M2 polarizing signal TGFβ, through the induced expression of kinase IRAK-M, an inactive serine/threonine kinase that acts as a negative regulator of TLR signaling (132). Of relevance, a recent report has associated M2-like macrophage infiltration with PD-L1 expression in gastric adenocarcinoma (133). Hence, as a key transcriptional component setting the onset and the resolution phase of inflammation, the different forms of NF-κB activation appear as the main regulators of TAMs functional heterogeneity, including their suppressive activity mediated by PD-L1.

## Concluding Remarks: Translational Implications

NF-κB, being a master regulator of inflammation, represents a link between immune response and cell growth (134). According to this view, inhibiting NF-κB signaling might counteract inflammation, tumor growth, and possibly reduce PD-L1 expression. NF-κB pathway is the primary or secondary target of different currently used drugs for the treatment of multiple myeloma (135). IKK inhibitors are commercially available and have been tested in preclinical studies for the management of different tumoral and inflammatory pathologies [for comprehensive reviews on the therapeutic implications of IKK targeting see (136, 137)]. Nevertheless, several concerns exist regarding the administration of NF-κB inhibitors due to their pleiotropic effects. To address this issue, current approaches comprise intermittent administration and use as adjuvant therapy. Alternative approaches that can be considered to reduce unwanted effects include either targeting components of the NF-κB pathway other than IKK or modulating NF-κB-dependent downstream effectors. In brief, NF-κB inhibition could be especially relevant in the context of cancer immunotherapies aiming to prevent PD-L1 overexpression and to modulate TAM survival/polarization.

Conversely, immunotherapy based on immune checkpoint inhibition (ICI) has revolutionized cancer treatment and PD-1/PD-L1 axis is the target of different monoclonal antibodies approved for human use (Pembrolizumab and Nivolumab are approved anti-PD-1; Atezolizumab, Avelumab, Durvalumab are approved anti-PD-L1). It is still unclear if they have different activity and/or toxicity. The majority of these compounds are engineered in order to prevent antibody-dependent cell cytotoxicity (ADCC), but Avelumab, an anti-PD-L1 IgG1 isotype, is able to perform both immune checkpoint inhibition and ADCC so that TAMs, MDSCs, and T_reg_, which can express high levels of PD-L1 in the tumor infiltrate, can be targeted as well as cancer cells (35, 138, 139). Immune-related adverse effects (irAEs) that resemble autoimmune responses occur during ICI therapy. Indeed, breakdown of the homeostatic PD-1/PD-L1 axis can provoke colitis, hepatitis, endocrinopathies, kidney injury, and skin problems (4, 140).

PD-L1 expression, as evaluated by immunohistochemistry, is routinely used as a biomarker for patient eligibility to anti-PD-1/PD-L1 therapy, and it is the only reliable molecular biomarker nowadays. Nevertheless, responsiveness to therapy does not mirror PD-L1 expression, and unfortunately a few PD-L1^+^ patients undergo hyper-progressive disease after ICI therapy (141–143). These discrepancies can be ascribed to several issues, including technical limitations of PD-L1 expression analysis, intra-tumoral heterogeneity, tumor mutational burden, inefficient priming of anti-tumoral T cells, and inadequate T-cell responses due to either tumor-intrinsic (compensatory immune checkpoints) or tumor-extrinsic (immune suppressive milieu) factors (144). It remains to be determined whether there are differences among individual drugs targeting PD-1/PD-L1 axis that are relevant for their clinical use and whether some patients would better benefit of either anti-PD-1 or anti-PD-L1 treatment. Although blocking either PD-L1 or PD-1 should similarly inhibit their molecular interaction, it is conceivable that, at least in some anti-PD-L1-treated patients, unblocked PD-L2 activity on PD-1 receptor can still inhibit anti-tumor response. Furthermore, considering that anti-CTLA-4 therapeutic efficacy can heavily depend on depletion of intra-tumoral CTLA-4^+^ T_reg_ by ADCC (145), it is possible that anti-PD-1/PD-L1 therapy efficacy could similarly depend on the depletion of either PD-1^+^ and/or PD-L1^+^ tumor-infiltrating T_reg_, at least in those patients in which these types of inhibitory cells dominate suppression of anti-tumor response. Accordingly, patient-tailored anti-PD-1/PD-L1 therapy is a subject of intense investigation [for example, see (146)].

It is conceivable that it is not PD-L1 expression *per se*, but rather the tumor microenvironment that induces PD-L1, that accounts for therapy success. A more comprehensive characterization of the tumor environment in terms of cytokine milieu, type of lymphocyte infiltration, macrophage phenotype would lead to improved approaches, most likely involving combined therapies (147, 148).

In this regard, the NF-κB state of activation, rather than PD-L1 alone, could have a prognostic value, as recently suggested by a detailed investigation of different types of human cancers, which reports how the local immune landscape drives clinical outcome (38). In patients with cancer, a positive or negative prognostic value has been correlated to the activation state of STAT1 and NF-κB pathways, respectively, despite the signaling of either being enough to lead to PD-L1 expression (38). It is tempting to speculate that the STAT factors, evolved in response to viral infections as components of the IFN-activated pathways that limit viremia, have anti-cancer features mostly by mediating pro-apoptotic effects. In contrast, NF-κB, evolved to regulate inflammation and tissue healing, and thus supporting cell survival and proliferation, drives pro-survival functions in cancer settings.

PD-L1 expression controls the strength of the immune response and acts as a rheostat of inflammation. Unfortunately, this mechanism is exploited by cancer cells to perform immune evasion. PD-L1 regulation during tumor progression evokes its physiological modulation, as discussed in the present review. In this scenario, uncovering the NF-κB-mediated regulation of PD-L1 in tumors can pave the way towards tailored therapeutic approaches targeting the PD-1/PD-L1 axis.

## Author Contributions

FA and FD searched for literature articles, conceived, and wrote the manuscript. AN, MG, ASi, and ASa revised and critically contributed to the manuscript drafting. FA, AN, MG, ASi, ASa, and FD approved the final version of the manuscript.

## Conflict of Interest

MG reports personal fees as consultant/member of advisory board from Eli Lilly, Boehringer Ingelheim, Otsuka Pharma, Astra Zeneca, Novartis, BMS, Roche, Pfizer, Celgene, Incyte, Inivata, Takeda, Bayer, MSD, GlaxoSmithKline S.p.A., Sanofi-Aventis, Spectrum Pharmaceuticals, Blueprint Medicine, Seattle Genetics, Daiichi Sankyo, Jannesen. The remaining authors declare that the research was conducted in the absence of any commercial or financial relationships that could be construed as a potential conflict of interest.
